# High Serum Erythropoietin and Ferritin Levels in Conjunction with Anemia Response in Malignant Lymphoma

**DOI:** 10.4084/MJHID.2011.018

**Published:** 2011-05-16

**Authors:** Sofia Omari, Alhossain Khalafallah, Mahmoud Ayesh, Ismail Matalka, Raji Al-Hadithi

**Affiliations:** 1 School of Human Life Sciences, University of Tasmania, Newnham, Tasmania, 7248, Australia; 2 Department of Pathology, Pathology & Laboratory Medicine Department, Jordan University of Science and Technology, King Abdullah University Teaching Hospital, Irbid, Jordan; 3 Department of Haematology, Launceston General Hospital, Tasmania, Australia; 4 Department of Internal Medicine, Jordan University of Science and Technology, Irbid, Jordan

## Abstract

Anemia is a common finding in lymphoma. There are few data available regarding the erythropoietin (EPO) levels in conjunction with ferritin in lymphoma patients. We prospectively evaluated 55 patients diagnosed with malignant lymphoma during the period between November 2006 and March 2008 at the King Abdullah University Teaching Hospital, Jordan. Our data showed that 74.4% of lymphoma patients were anemic. Furthermore, serum EPO and ferritin levels were higher in lymphoma patients compared with the healthy controls (P=0.001). The observed versus predicted EPO ratio showed also significantly higher levels in anemic lymphoma patients compared to healthy controls (p=0.03). There was an improvement in the Hb level in lymphoma patients who were treated with at least 3-cycles of chemotherapy as compared with newly-diagnosed patients. An adequate increase of EPO levels was observed in anemic lymphoma patients and notably associated with higher ferritin levels and improvement of Hb (p<0.001).

Our findings suggest that ferritin estimation in lymphoma patients may predict the level of erythropoiesis and possibly the degree of anemia. Further studies to confirm these findings are warranted.

## Introduction:

Anemia is common in patients with cancer, especially Hodgkin’s lymphoma (HL) and non-Hodgkin’s lymphomas (NHL).^1^,^2^ About 30–40 % of lymphoma patients present with anemia prior to commencement of chemotherapy. The anemia is mainly attributed to bone marrow replacement by lymphoma cells or inadequate erythropoietin (EPO) production which leads to suppressed erythropoiesis.^3^ The frequency of anemia increases in all malignancies, with the largest increase in patients with NHL (from 35.1% at baseline to 73.7%) and HL (from 21.9% to 54.5%) under the age of 60.^4^–^6^ EPO stimulates the proliferation and maturation of erythroid precursors in the bone marrow. The earliest progenitor form that can be identified is the erythroid burst-forming unit (BFU-E), which subsequently evolve into erythroid colony-forming units (CFU-E). EPO acts primarily on these cells by stimulating cell division and maturation as well as inhibiting apoptosis.^7^ Tissue hypoxia triggers endogenous EPO production when hemoglobin (Hb) falls below 12 g/dL.^8^,^9^ When hypoxia resolves, EPO production decreases.^9^

Initial studies in patients with cancer found that most of the patients had disproportionately low levels of endogenous EPO for the degree of their anemia, especially after chemotherapy.^10^–^12^ Some studies showed that cancer-related anemia patients had serum EPO levels above the normal range which shows their strong ability into self-body regulation in response to anemia.^10^–^12^ Although some studies suggested that EPO levels decrease in malignant lymphoma,^11^,^13^ however, others reported that EPO levels increase in anemic lymphoma patients, indicating that anemia did not depend on defective EPO secretion.^14^ Ferritin is synthesised and released from malignant lymphocytes in a faster manner than normal lymphocytes causing ferritin concentration to increase in those patients.^15^–^17^ However, there is very few studies that address EPO response in association with ferritin levels in conjunction with anemia response in malignant lymphoma. The aims of our study were to determine (a) the EPO and ferritin levels in *de novo* and treated lymphoma patients, and (b) whether there is a relationship between EPO level and ferritin response to anemia.

## Patients and Methods:

Fifty-five serum samples were obtained from patients who were diagnosed with malignant lymphomas (29 HL and 26 NHL) during the period between November 2006 to March 2008 at the King Abdullah University Teaching Hospital. Written consent was obtained from all patients. This study was ethically approved by the Conjoint Ethics Committee of Jordan University of Science and Technology. Thirty samples were obtained from newly-diagnosed patients and twenty-five from patients who were receiving chemotherapy after at least 3 cycles of chemotherapy. Both groups included anemic and non-anemic patients. The specific diagnosis and the stage of the disease for each lymphoma patient were obtained from patients clinical notes. We included in the study newly diagnosed patients with malignant lymphoma and patients who had received at least three cycles of chemotherapy for malignant lymphoma and had not received blood transfusions for at least 12 weeks.

There were an equal number of samples collected at the time of diagnosis and after 3 cycles of chemotherapy. Bone marrow studies were performed for all patients and showed that 13 cases had bone marrow involvement (stage IV lymphoma disease) without a significant compromise of the hemopoeitic reserve.

We excluded patients with hepatitis, infections, liver disease, renal failure, iron, B12 and folate deficiencies and patients with hemolytic anemia as these conditions may influence the levels of ferritin and EPO. Infections markers were negative for the patients included in the study. Furthermore, no patients received erythropoietin therapy in this study.

Fifty-five healthy, sex and age-matched volunteers were randomly selected as a control group. All control group participants provided a written consent.

Non-fasting 5–10 ml serum samples were obtained from all participants during the morning at room temperature. Samples were centrifuged within 30 minutes of collection at 2000 rpm for 10 minutes at room temperature and sera were isolated from each sample. For each patient, the following tests were performed: serum EPO, Hb, creatinine, lactate dehydrogenase (LDH), B12, folate and ferritin. Serum vitamin B12 was obtained to exclude megaloblastic anemia. EPO was measured by ELISA (IBL-Hamburg kit, Germany). Ferritin levels were measured by electrochemiluminescence immunoassay (ECLIA) using an Elecsys 1020-immunoassay analyzer (Roche Diagnostics, Indianapolis, Indiana, USA).

### Definitions:

Stage A lymphoma disease is defined by absence of constitutional symptoms, while stage B means presence of B-constitutional symptoms such as weight loss, night sweats or fever.

Stage I–II lymphoma represent a localized nodal disease, while stage III lymphoma disease represents wide-speared nodal disease but with absence of non-reticuloendothelial organ involvement, and stage IV lymphoma disease reflects organ involvement such as bone marrow, liver or lung involvement. Extra-nodal disease is defined as involvement of other organs that are not related to the reticulo-endothelial system. Severe anemia is defined by a level of Hb <8 g/dl, while moderate anemia; Hb=8–10 g/dl and mild anemia in males; Hb=10–13 g/dl and in females; Hb=10–12 g/dl.

### Statistical analysis:

Means, standard deviations (SD) and difference of means were estimated for illustrative purposes using general linear modelling. Assumptions of linear regression were violated in most of these analyses due to heteroskedasticity and skewness of residuals; therefore, non-parametric analyses (ordinal logistic regression) were performed to test differences of distributions. P-values were corrected for multiple comparisons where appropriate by the Holm method. All statistical analyses were performed using Stata/SE 11.0 (StataCorp, College Station, Tx USA).

Observed/Predicted (O/P) EPO ratio was calculated in order to determine the endogenous erythropoietin response to the level of Hb in our cohort of patients while at the same time avoiding possible interference by the endogenous erythropoietin production and/or erythropoietin activity.

The O/P EPO ratio has been calculated by dividing the observed log erythropoietin levels of the patients by the predicted log erythropoietin levels to obtain the O/P ratio. The same ratio has been also calculated for the control group.

## Results:

We estimated serum EPO levels in 55 normal healthy controls where the EPO mean was 7 mU/L (SD± 5.3). We observed that lymphoma patients had higher EPO levels compared with the healthy control group. Hb was significantly lower in lymphoma patients compared with the control group (P<0.001). Lymphoma patients had significantly higher ferritin levels than the control group (P<0.001; Table 1). Also, Hb was lower in newly-diagnosed lymphoma patients when compared to the lymphoma patients in the chemotherapy group (P=0.003). EPO, LDH and ferritin concentrations were similar for newly-diagnosed lymphoma patients and patients who underwent chemotherapy. Stage B lymphoma (symptomatic) patients comprised most of the lymphoma study group (73.9 %) compared with 19.6 % for stage A lymphoma (asymptomatic) and 6.5% for extranodal involvement patients. Stage A lymphoma patients had significantly higher Hb concentrations compared with those with stage B (P=0.028). However, EPO levels were not significantly higher in stage B lymphoma patients compared with stage A patients (P=0.4), and there were no significant differences observed in LDH and ferritin levels between stage A and stage B lymphoma patients. There was no statistical difference seen between HL and NHL patients in regard to EPO, Hb, ferritin, and LDH levels.

Anemic patients accounted for 74.4% of the sample patients while non-anemic patients for 25.6%. There were 4 cases who had severe anemia and 19 had moderate anemia and 18 had mild anemia, while 14 patients were not anemic.

**Figure 1.**
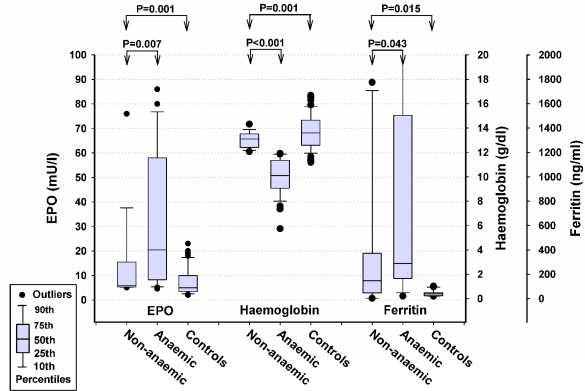
Distributions of non-anemic and anemic lymphoma patients compared with normal healthy controls

Anemic patients had higher levels of EPO than non-anemic patients or healthy controls (P=0.001). However, the observed/predicted EPO ratio after adjustment for endogenous EPO response from the normal EPO production/activity continue to demonstrate significantly higher levels in anemic lymphoma patients versus healthy controls (p=0.03) (Table 1). There were significantly higher ferritin levels in non-anemic lymphoma patients compared with the healthy control group (P=0.015; Figure 1). EPO, Hb and ferritin levels were significantly higher in non-anemic lymphoma patients compared to healthy controls.

Hb levels were significantly lower in “high EPO group” compared to the “low-normal EPO group”.

## Discussion:

Our analysis showed that serum EPO levels were higher in lymphoma patients compared with the healthy control group. This is most likely due to EPO response to anemia in lymphoma patients. Furthermore, anemic lymphoma patients (74.4%) had higher EPO levels compared to non-anemic lymphoma patients. Similar findings have been reported by Birgegård *et al*;^5^ Li *et al*^12^ and others.^18^ Serum EPO concentrations in lymphoma patients exhibited wide variation, ranging from normal levels to high levels in association with anemia. Some studies showed a decreased level of EPO in treated lymphoma patients,^3^,^11^,^13^ whereas the present study demonstrats no difference in EPO levels between newly-diagnosed lymphoma patients and patients on chemotherapy. Our results are consistent with a previous study reported by Ozguroglu *et al*.^18^ However, Lee^11^ reported low levels of EPO in patients receiving chemotherapy compared with newly-diagnosed patients. An inverse relationship between Hb level and EPO was seen in our series in accordance with other studies.^3^,^18^ Furthermore, the current study showed an improvement in Hb level in lymphoma patients treated with chemotherapy for at least three cycles as compared with patients who were newly-diagnosed (P=0.003). This is largely due to the fact that most of the treated patients for lymphoma disease were in advanced clinical stage III–IV (65%) that has responded with improvement of Hb secondary to the commenced treatment. Furthermore, stage A lymphoma patients had significantly higher initial Hb levels compared with stage B patients (P=0.028). Higher EPO levels were found in stage B patients in comparison with stage A but was not statistically significant. We observed higher ferritin levels in lymphoma patients with high EPO levels compared to healthy controls (P<0.001), that has been explained by erythropoietic response to anemia. Therefore, the ferritin level may indicate the increase of EPO activity among lymphoma patients.

## Conclusions:

In summary, an adequate EPO and ferritin response to anemia in patients with lymphoma was demonstrated in our study. This may indicate that recombinant human EPO (rhuEPO) treatment is not required in anemic lymphoma patients who have acceptable EPO and ferritin response. Results obtained from this study would help in understanding different factors that contribute in the degree of anemia among lymphoma patients. Furthermore, most of the patients with high ferritin levels had significantly high EPO levels. Perhaps more studies can address utilization of ferritin as a possible marker for EPO activity in addition to measuring the response to anemia in this cohort of patients. Of note, the cost of ferritin measurement is much lower than that of EPO. Moreover, ferritin testing is more accessible in most laboratories compared to EPO. However, one of the important limitations is that ferritin level can be affected as an acute phase reactant by the degree of infection or inflammation that may coincide in lymphoma patients. It is worth noting that we excluded all patients with infections from the current study. Nevertheless, further studies to confirm our findings are warranted.

## Figures and Tables

**Table 1.** Characteristics and laboratory results of the patients and healthy controls

	**Normal range**	**Healthy controls**	**Lymphoma patients**	**Comparison**		
		**(N=55: Male 31) Mean (SD)**	**(N=54: Male 25) Mean (SD)**	**Difference**	**95% CI**	**P-value**
**Age (years)**		43.6 (1.9)	40.7 (14.2)	−2.9	(−6.8 to 1.0)	0.14
**EPO level (mU/l)**	3–20	7.0 (5.3)	26.2 (25.8)	19.2	(12.2 to 26.2)	<0.001
**O/P EPO ratio**		1.70 (2.00)	1.04 (1.29)	−0.66	(−1.29 to −0.04)	0.038
**Hb (g/dl)**	14–18 (male)	13.7 (1.4)	11.0 (1.9)	−2.7	(−3.3 to −2.1)	<0.001
	12–16 (female)					
**Ferritin (ng/ml)**	20–300	43 (24)	627 (729)	583	(359 to 808)	<0.001

EPO, serum erythropoietin. Hb, hemoglobin. Mean (SD), and difference estimated by general linear modeling for illustrative purposes, and comparison tested using Mann-Whitney rank-order test due to non-normal distributions. O/P, observed/predicted EPO ratio after adjustment for endogenous EPO response from the normal EPO production/activity.
